# Supplementary material for: Are carbon emissions trading and green financial instruments synergistic? -Comprehensive quantitative research based on content analysis
Source: PLoS One. 2024 Mar 7;19(3):e0298601. doi: 10.1371/journal.pone.0298601 (PMC10919601; doi:10.1371/journal.pone.0298601)
Supplement: S1 Data — (ZIP) [file pone.0298601.s001.zip › Data/地级市Z_F工作报告.zip!/╣π╢1⁄2/├»├√╩╨/└┤╫╘╣1⁄2╓┌║┼í╛┬φ┐╦╩2╛▌═°í┐_2018.docx]

各位代表：

　　我代表市人民政府向大会作工作报告，请予审议，并请政协各位委员和其他列席人员提出意见。

　　2017年工作回顾

　　过去一年，是本届政府依法履职的第一年，也是茂名凝心聚力、厚积薄发的重要一年。在省委、省政府和市委的坚强领导下，在市人大及其常委会和市政协监督支持下，市政府坚持以习近平新时代中国特色社会主义思想为指导，深入贯彻落实党的十八大和十八届历次全会精神，认真学习贯彻党的十九大精神，牢牢把握稳中求进工作总基调，坚持不懈抓重点、补短板、强弱项，坚定不移稳增长、促转型、惠民生，较好完成了十二届人大一次会议确定的经济社会发展任务。

　　（一）着力推进结构调整，发展质量和效益不断提升

　　综合实力持续增强。2017年，全市实现地区生产总值2924.21亿元，增长7.5%，经济总量继续位居粤东西北首位。完成固定资产投资1415.73亿元，增长12.1%。一般公共预算收入130.14亿元，增长7.5%。完成规上工业增加值873.47亿元，增长7.1%；工业经济效益综合指数同比提高42.6个百分点。全年贷款余额增长14.0%，存贷比46.7%，同比上升1.3个百分点。城镇和农村常住居民可支配收入分别增长8.5％、8.1％，均高于GDP增速。全市单位GDP能耗同比下降4.2％，环境质量持续改善。在省对粤东西北2016年振兴发展评估考核中获优秀等次。

　　产业结构趋于优化。六大主导产业增加值787.63亿元，占全市比重90.3%。旅游业总收入增长35.5%，装备制造业增加值增长25.4%。全市市场主体突破20万户，新登记注册企业增长43.1%。20万吨/年环氧乙烷、露天矿林光互补等项目建成投产。与中国恒天、中国能源、中国建材、协鑫集团、正大集团等知名企业加强务实合作。民营经济成为地方经济主力军，约占全市经济比重62%。全市实现省级工业园区全覆盖。三次产业比重调整为15.6：40.1：44.3，二三产业占比提高1个百分点。

　　创新驱动成效明显。全社会R&D投入17.8亿元，获省科技奖8项，均居粤东西北首位。新增省级工程技术中心21家。高新技术企业有望突破110家。申报高新技术产品133个，为历年最多，一批新产品、新技术从茂名走向市场。广东众和成功获评省政府质量奖，实现粤西企业零的突破。

　　（二）着力推动基础先行，厚植茂名发展优势

　　交通建设再掀新高潮。全年完成交通投资109亿元，增长44.3%。汕湛高速茂名段建成通车，有效疏解通往珠三角交通拥堵。云茂高速全面建设。谋划建设中山至高州（粤桂界）高速公路。深茂铁路茂名段即将完工。茂名综合客运枢纽项目、博贺疏港铁路即将动工建设。市域组团之间初步形成“六纵三横”快速干线路网，西部快线、工业大道南、潘州大道和S291线改建工程完成路面工程，南排大道、中德大道、茂化快线东延线开工建设，广东滨海旅游公路茂名段加快前期工作。

　　环保设施建设有序推进。化州绿能环保项目动工建设，电白、信宜项目已完成招投标，高州项目加快推进前期工作。城乡水质净化设施扎实推进，电白获批全省整县推进生活污水处理设施建设示范区。市政污水管网建设快速推进。城乡清洁工程长效机制逐步建立。

　　水利等民生设施建设取得新进展。中小河流治理提速发力。河东水厂三期扩容工程推进顺利。滨海新区供水工程如期推进，水东湾引罗供水工程即将竣工。全市电力输配网络趋于完善，信息、邮政网络覆盖范围进一步拓展。

　　（三）着力推动城市进步，焕发城市发展新活力

　　“双创”工作全面启动。致力让城市更美好，全面启动创建全国文明城市和国家卫生城市。参加央视《魅力中国城》竞演获得巨大成功，荣获“十佳魅力城市”和“最受观众喜爱魅力城市”称号，深度挖掘了城市特色文化，整合了文化旅游资源，凝聚了民心民力，增强了城市自信，树立了“好心茂名”城市形象，为城市文明进步注入新动力。

　　城市建设展现新气象。北组团五个片区全面开发建设；建成站前大道、文明北路、大园三路，加快建设金城路、大园一二路、人民北路等12条城市道路，完成30多项道路整治和升级改造工程。中组团共青河新城城市设计和控制性详细规划正在编制。南组团大力推进“八路一堤一广场”建设。完成包茂大道南、水东大道、环市路、迎宾北路新建（或扩建）工程。

　　城市人居环境提质升级。建成官渡公园、上宾体育公园、江东体育公园及1号湖公园，小东江十里休闲景观带初步打造为城市会客厅。多个城市综合体落地建设。超额完成省定黑臭水体整治任务。推进交投集团与广东粤运公司战略重组，加快公共交通发展。电白区精心打造东西两湖、海堤栈道景观，荔枝公园即将建成，水库公园、沉香文化公园开工建设。

　　新型城镇化加速推进。加快完善全域城乡建设规划。推动电白沙琅、信宜钱排、高州长坡、化州合江建设县域副中心。沙琅镇入选全国特色小镇，马贵镇入选省首批特色小镇示范点。出台户籍制度改革意见。

　　（四）着力推动平台发展，向海而兴迈出新步伐

　　滨海新区起步区加速发展。防波堤工程接近完工，10万吨航道工程基本完成。广州港通用码头建设顺利推进，粤电煤炭码头基本完成主体工程。粤西LNG造陆工程开工建设。博贺湾新城东片区土地一级开发、新城东路等项目加快推进。

　　高新区进入发展新阶段。创建国家高新区材料已报国务院。获批国家知识产权试点园区，高新区科技企业孵化器晋升国家级，圆梦创客成为国家众创空间。30万吨粤桥钛矿等14个项目建成投产，应急救援基地等49个项目顺利推进。

　　水东湾新城建设步入快车道。高标准规划环湾城市景观风貌带。规划建设南海旅游岛“五大公园”，水东湾海洋公园、歌美海公园（一期）完成建设。国际会展中心、市中心医院（一期）主体工程基本完成。南海旅游岛·中国第一滩景区获评国家4A级旅游景区。

　　（五）着力建设农业强市，农业农村现代化取得新进展

　　现代农业成果丰硕。省级以上农业龙头企业达50家，居全省第三。全市农业企业推进产学研合作项目300多项。新建省级农业标准化示范区15个，建成高标准农田28.5万亩。新型农业经营主体突破1万个。省级农产品加工示范园获批建设。

　　新农村建设加快推进。村村通自来水工程完成投资5.4亿元。全市行政村保洁覆盖率达97.6%、客运班车通达率93.58%。农村土地确权登记颁证加快推进。精准扶贫进展顺利，帮扶项目45.82万个，预计全年脱贫5.9万人。完成1.53万户农村危房改造。省定贫困村创建新农村示范村全面铺开。

　　（六）着力加强环境治理，生态环境持续改善

　　环境综合治理成效明显。空气质量指数稳居全省第三。全面推行河长制。鉴江、小东江和高州水库等重点区域综合污染指数持续下降。城市集中式饮用水水源水质达标率100%。近岸海域水质保持稳定。农业面源污染、土壤污染防治扎实推进。完成1056家禁养区内养殖场关闭任务。淘汰黄标车10387辆。严厉打击环境违法行为，立案查处环境违法企业250宗。

　　国家森林城市建设进入新阶段。扎实推进新一轮绿化茂名大行动。完成造林更新面积24.6万亩，打造森林家园70个，创建生态示范村18条，在东北部山区打造百里银杏长廊。国家森林城市建设总体规划通过专家评审。露天矿生态公园被评为国家矿山公园。信宜钱排镇、高州根子镇被认定为省森林小镇。

　　（七）着力深化改革开放，内生发展动力有效增强

　　行政体制改革取得新进展。“放管服”改革纵深推进。“一门式一网式”改革更加深入，新的市行政服务中心建成运作。网上办事服务大厅市级办结率90%以上。实现全市村（社区）公共服务全覆盖。有效推进城市执法体制改革。深化商事制度改革。纪检派驻机构实现全覆盖。完成羊角镇区划调整工作。

　　经济体制进一步优化。供给侧结构性改革成效明显，取缔“地条钢”产能78万吨，减轻企业负担20.3亿元。推进与中国高岭土公司等大型企业战略合作，城投集团组建工作取得重大进展。信宜市农村信用合作联社成功改制为农村商业银行。中信银行在茂名设立分支机构。高州长江村镇银行开业。

　　开放合作不断深化。积极融入北部湾城市群。对标学习惠州，加强与广西玉林、梧州和黑龙江伊春合作。主动融入“一带一路”建设，深化与德国、荷兰、日本等国家地区交流合作。加入中德经济顾问委员会、中德工业城市联盟，牵头成立中德工业城市联盟石化专委。经济外向度不断提高，全年实现进出口总额增长29.1%，增幅居全省首位。

　　（八）着力增进民生福祉，努力让群众生活更美好

　　民生实事稳步推进。民生类支出321.6亿元，占财政支出84.2%，同比增长8.1%。省十件民生实事配套资金支出进度连续四年排全省第一。新增城镇就业7万人，新增转移就业劳动力5万人，官渡街道沿江社区被认定为国家充分就业社区。建成省粤西创业创新孵化基地。城乡低保、五保救助、残疾人等弱势群体保障水平大幅提高。投入6亿元推进茂名石化卫生防护距离搬迁安置工作。建成各类保障性住房和棚户区改造住房1285套，新开工棚户区改造住房790套。筹集住房公积金32亿元支持职工改善住房条件。

　　文教卫生事业成绩斐然。全面推进教育创现工作，建成育才学校，动工建设崇文学堂，中心城区新增学位2751个。华南师大附属电白学校合作办学正式签约。广东石油化工学院西城校区、广东农林科技职业学院开工建设。信宜荣获“国家级农村职业教育和成人教育示范县”称号。成功举办南海开渔节暨省岭南民俗文化节、南国书香节茂名分会场等活动。圆满举办“好心茂名”龙舟邀请赛、市第十三届运动会和市长杯足球赛，积极申办省运会。在省第七届群众音乐舞蹈花会的比赛成绩居粤东西北首位。全面推进卫生创强，启动城市公立医院综合改革。高州被定为全国医改示范县。

　　社会治理深入推进。加强基层派出所建设。严厉打击涉枪、涉毒、涉电信诈骗、涉金融领域突出违法犯罪，电白区电信网络诈骗犯罪重点地区成功摘帽，社会治安明显好转。深入开展平安社区创建活动。全力推进平安建设“红袖章”工程和“雪亮工程”。抓好危险化学品、道路交通、建筑施工等领域安全管理，全年未发生较大及以上生产安全事故，安全生产形势保持稳定。食品安全检测能力建设项目顺利完工。全面完成村（居）换届选举工作。同时，人口计生、工青妇、民族宗教、国防动员、残疾人、统计、双拥、气象、地震、外事、侨务、三防、人防、打私、打假、地方志、水库移民等工作都取得新成绩。

　　我们着力加强自身建设，打造务实清廉担当的政府形象。办理人大代表建议116件、政协委员提案289件。扎实推进政府立法，起草完成冼夫人文化遗产及露天矿生态公园相关保护条例草案。健全政府法律顾问制度，全省依法行政考评保持良好等次。全面推进政务公开工作。落实全面从严治党主体责任和监督责任。制定实施加强和改进政府机关作风工作方案，行政效能有效提升。推进“两学一做”学习教育常态化制度化。深化廉洁政府建设，不断强化监察、审计监督作用。

　　各位代表！过去一年成绩来之不易。这是省委、省政府和市委正确领导的结果，是全市人民团结拼搏、共同奋斗的结果。在此，我代表市人民政府向全市人民，向驻茂人民解放军、武警官兵和各民主党派、工商联、人民团体、社会各界人士致以崇高敬意！向长期关心支持我市发展的港澳同胞、台湾同胞、海外侨胞及国际友人表示衷心感谢！

　　我们也清醒地认识到，我市改革发展中还有不少需要破解的难题：营商环境有待改善，招商引资有待突破，实体经济发展水平有待提高；个别区（市）隐性债务规模较大；城市建设管理水平不高，中心城区集聚能力较弱；机关作风仍需改进，少数干部干事创业精气神不足；民生领域欠账多，扶贫攻坚任务重；生态环境治理压力较大；发展所需各领域专业人才缺乏。对这些问题，我们将采取有力措施逐步解决好。

　　2018年工作安排

　　各位代表！今年是全面贯彻党的十九大精神的开局之年，是改革开放40周年和迎接茂名建市60周年冲刺之年，做好今年工作意义十分重大。我们要把学习贯彻十九大精神与落实习近平总书记对广东工作重要指示批示要求贯通起来，与推动茂名振兴发展紧密结合起来，坚决贯彻落实习近平新时代中国特色社会主义经济思想，进一步完善我市发展思路举措和政策措施。一是更加注重融入国家和省的战略规划。牢牢把握十九大提出系列发展战略带来的重大机遇，落实建设海洋强国战略和省沿海经济带综合发展规划，争创海洋经济发展示范区；实施创新驱动，继续在粤东西北创新发展方面走在前列；实施乡村振兴战略，加快农业大市向现代农业强市迈进；积极融入“一带一路”建设，加强与沿线国家和地区合作；落实《北部湾城市群发展规划》，努力在区域经济发展中占据更有利位置。二是更加注重高质量发展。大力推进供给侧结构性改革，把创新作为引领发展第一动力，打好大气、水、土壤污染防治“三大战役”，提升发展质量效益，改善生态环境，增强群众获得感。三是更加注重特色发展。抓住深茂铁路开通和全省一体化发展机遇，依托海洋、化工、农业、矿产、劳动力、气候等优势资源，发挥好“三大平台”和省级园区等载体作用，在更大区域范围内集聚发展要素，努力建成一批特色产业集群。四是更加注重实体经济。深刻认识茂湛炼化一体化基地加快建设和新能源产业蓬勃发展对我市产业未来发展的影响，加快石化产业转型升级，加大招商引资力度，拓展招商引资领域，实现实体经济由一业独大到多点支撑的转变。五是更加注重文化引领。充分用好“魅力中国城”竞演效应和“双创”平台，大力弘扬“好心精神”，充分发挥地方特色文化引领作用，进一步凝聚共识、形成合力、树立品牌。六是更加注重实干兴茂。坚持干字当头、问题导向、重点发力、以上率下，实施好建市60周年系列行动计划，集中攻坚一批重难点项目，夯实我市发展基础。

　　2018年政府工作的总体要求是：全面贯彻落实党的十九大和中央经济工作会议精神，以习近平新时代中国特色社会主义思想为指导，认真贯彻习近平总书记对广东工作的重要指示批示、省委十二届三次全会和市委十一届六次全会精神，围绕全面建成小康社会目标，坚持稳中求进工作总基调，坚持新发展理念，贯彻高质量发展要求，统筹推进“一个总体目标、三个阶段性目标、六个致力打造”，培育壮大实体经济，深入推进改革创新，加强基础设施建设，推动城市扩容提质，突出文化引领，切实保障和改善民生，促进绿色发展，凝心聚力加快茂名振兴发展。

　　今年经济社会发展主要预期目标是：地区生产总值增长7.0 %左右，固定资产投资增长10.0%，进出口总值、利用外资完成省下达任务，社会消费品零售总额增长10.0%，地方一般公共预算收入增长6.0%，研发经费支出占地区生产总值比重达0.66%，居民消费价格涨幅控制在3.0%以内，居民收入增长速度高于经济增长，单位生产总值能耗、主要污染物总量和大气、水环境质量完成省下达的目标任务。

　　今年重点要抓好以下九项工作：

　　（一）大力发展实体经济，建设现代化经济体系

　　壮大工业主导产业。实施《培育壮大工业主导产业五年行动计划》和《迎接建市60周年产业行动计划》，开展“走听看想比”活动。力争茂名石化150万吨/年连续重整装置、天源石化丙烯、长青热电联产等项目建成投产，茂名石化炼油产品结构优化项目、和标轻烃综合利用、电白波顿香精香料等项目开工建设。推进成品油储备库、茂名石化苯乙烯装置和博贺新港区至炼油厂油品管道工程等项目前期工作。加快电白粤西中药港、高新区大参林生产基地、化橘红产业园等项目建设。

　　培育新业态新动能。争取新兴产业布局茂名，大力发展新能源、新材料、大数据、装配式建筑等产业。力争中机信息（茂名）数据中心建成投产。推动氢能源高新技术研发中心和氢燃料电池产业示范基地项目尽快落地。推动粤西LNG接收站项目取得实质性进展。大力发展滨海度假、山地养生、乡村休闲、文化体验四大旅游板块，支持电白、信宜建成全域旅游示范区（市）。积极发展医疗保健、养老康复、运动健身等新业态，加快市中心医院、市中医院新院区和配套健康养老项目建设。

　　促进产业平台提质增效。引导园区由一般性综合性园区向专业化园区发展。实现省级产业转移园承接珠三角产业梯度招商项目30个。成功创建国家高新区，推进中德（茂名）精细化工园建设。动工建设高岭土产业园，规范发展高岭土产业。启动建设南方国际汽车产业园。加快博贺新港区综合物流园、粤西（化州）空港经济区规划建设。支持六韬珠宝创意产业园发展。谋划特色小镇、产业综合体和田园综合体等产业平台。

　　加大招商引资力度。深刻认识工作主业与招商引资相互促进、共进共赢关系，着力形成全市一盘棋、上下联动、全行业全领域招商引资新格局。强化大项目招商规划，坚持把优势资源用于引进有理念、有经验、有实力、能发挥龙头带动作用的企业。加强招商队伍建设，完善招商引资优惠政策。建立健全招商引资工作绩效考核机制。定期举办精细化工、农产品深加工、基础设施、城市运营、旅游开发、医疗康养等领域精准招商活动。全面落实省、市“实体经济十条”和省“外资十条”。支持中小微企业发展。树立“亲则两利、清则两安”理念，积极构建“亲”清”新型政商关系。

　　积极发展开放型经济。推进湛茂阳沿海经济带建设。加强与粤港澳大湾区对接合作，深化对标学习惠州。深化与北部湾城市群城市合作。密切与黑龙江省伊春市对口合作关系。加快口岸服务中心建设。推进与德国、荷兰、俄罗斯、新加坡、意大利和日本等经贸合作，开拓“一带一路”新兴市场。

　　（二）大力推进改革创新，加快培育发展新优势

　　深入推进基础性关键性改革。推进“放管服”改革，开展“减证便民”行动，深入实施“多证合一、一照一码”登记制度改革。贯彻落实市场准入负面清单工作。争创省“数字政府”建设试点市，攻坚国家级政务服务标准化试点验收工作。全面铺开零基预算改革。推进国有企业重组合作。支持创新金融产品和服务方式。推动农村信用联社改制农商行。力争年内新增2家村镇银行。全力守住不发生系统性金融风险的底线。完成城市综合执法体制改革。突出抓好国家监察体制改革。建立健全公务员绩效考核体系。完成农村土地承包经营权确权登记颁证工作。开展第四次全国经济普查。贯彻落实中央、省农村土地“三权分置”实施意见，稳步推进农地经营权抵押贷款试点。积极探索农村“资源变股权、资金变股金、农民变股民”发展新模式。

　　切实提升创新能力。推动规上企业加大研发投入，建设一批高水平研发机构。新培育一批高新技术企业。支持广东石油化工学院建设高水平理工大学。加强与签约高校、科研院所产学研合作。争取通过省质量强市示范市验收。加强知识产权服务机构建设，建立知识产权维权援助中心，加快推进省市共建石化产业知识产权强市。出台我市深化人才发展体制机制改革的实施意见，完善高层次人才引进培养和奖补政策，加快高层次人才公寓建设。建设一批工程技术中心、院士工作站、博士后工作站。加快建立三级人才驿站服务体系。推进市人力资源服务产业园建设。完善高层次人才数据库。

　　（三）大力加强基础设施建设，改善发展硬环境

　　持续抓好交通基础设施建设。推动深茂铁路茂名段如期通车。完成茂湛铁路茂名段电气化改造。加快茂名综合客运枢纽和博贺疏港铁路建设。配合做好广湛高铁茂名段前期工作。谋划城市轨道交通。开工建设沈海高速茂名段扩建工程。加快云茂高速茂名段建设。推进中山至高州（粤桂界）高速公路规划建设。加快中德大道、南排大道、茂化快线东延线等市域路网建设。完成S291线高州石仔岭至茂南公馆段、S284线播扬至合江段路面改造工程，加快推进S291线高州云潭至电白黄岭改建工程。全力推进玉茂一级公路建设。

　　加快补齐基础设施短板。加快滨海新区供水、高州水库除险加固恢复89.0米正常蓄水位淹没处理项目、海堤达标加固等工程建设。持续抓好中小河流治理工程。加快电网、信息基础设施建设。协调推进充电桩建设。推动“厕所革命”。

　　加强道路交通管理。持续抓好城乡道路沿线“两违”整治。突出抓好超载超限违法运输、危险品运输、道路客运安全管理及涉渔“三无”船管理。加强公路养护管理。

　　（四）大力推进扩容提质，优化城市人居环境

　　加快城市建设步伐。开展北组团茂东快线和茂名大道微改造，实施“二河三路”（低埒河、隔坑河和茂南大道、西粤南路及站北六七路）截污工程，推进6个社区路网升级改造，建成西粤北路、江东北路、钟鼓西路、高凉南路、大园桥等“十路一桥五公园”，完成20条道路沥青罩面改造工程，继续推进城区亮化工程。稳步推进茂名石化炼油厂卫生防护距离内居民搬迁安置。抓好高新区国家新型城镇化试点工作。加快紫荆大道、崇文学堂、雨公山森林公园等项目建设。规划建设共青河新城。抓好电白城区“八路一堤一广场”市政建设，加快水霞路、凤凰大道、环市东路建设。大力加强城市公共交通建设。加快推进“三旧”改造工作。

　　大力发展城市经济。大力引进城市开发战略合作伙伴，进一步完善城市功能，丰富城市业态，繁荣城市经济。抓好万达广场、城光世纪城、保利中环广场、爱琴海购物公园等城市综合体建设。加快站南片区CBD规划建设，打造进入城市“第一印象区”。着力培育建筑业“总部经济”。推动特产、餐饮、水果、夜市等特色街区建设。

　　提升城市精细化管理水平。加强城管委统筹协调作用。推行网格化管理，加强社区治理、小区物业和农贸市场管理。推进园林绿化、清洁卫生市场化管理，持续抓好市容整治，逐步解决停车难问题。加快智慧城市建设。

　　全力推进“双创”工作。深入开展基础设施、市容市貌、城市管理、城市交通、城市安全、市民素质提升六大行动。打造若干城市文明示范小区。持续深入推进爱国卫生运动。

　　（五）大力发展海洋经济，推动陆海统筹发展

　　加快推动滨海发展战略。紧抓省支持东西两翼沿海经济带建设机遇，整合陆海资源要素，优化陆海产业布局，促进海岛岸湾港业城一体化发展，打造海洋经济强市。完成博贺新港区防波堤配套工程建设。加快建设粤电煤炭和广州港通用码头。推进水东湾环湾交通体系建设，全面开工建设广东滨海旅游公路茂名段。完善滨海新区产业规划修编。开工建设滨海新区电城产业园。围绕博贺渔港、放鸡岛、浪漫海岸、中国第一滩、水东湾、歌美海，打造“六点一线”南海博贺滨海旅游度假区。开工建设浪漫海岸海昌海洋公园。加快推进博贺湾新城建设，开工建设华南师大附属电白学校和茂名滨海学校，推进博贺渔港沿岸建筑立面改造及环境整治提升工程。支持博贺打造民宿创意小镇。着力推进歌美小镇项目。加快高地智慧城项目建设。

　　大力发展特色县域经济。立足县域特色资源优势，做大做强做精做深主导产业。茂南突出精细化工、高岭土加工、商贸物流和新兴产业，电白突出珠宝加工、香精香料、食品加工、家居建材和医药健康，信宜突出矿产资源、毛纺、玉器和特色农产品加工，高州突出“三高”农业、农产品加工、金属制品、纺织制衣和皮革制品，化州突出金属加工、医药健康、农副产品和矿产资源加工。加快编制镇村规划。统筹推进县域副中心规划建设。加快沙琅、马贵等特色小镇和钱排、根子等森林小镇建设发展步伐。

　　（六）大力实施乡村振兴战略，加快新农村建设

　　完善农村基础设施。突出抓好“四好农村路”。推进新一轮农村电网改造升级工程。完成村村通自来水工程建设任务。加快高标准基本农田建设，以“补改结合”方式落实耕地占补平衡。深化农村环境综合整治，推进“三清三拆三整治”。积极推进省级新农村示范片建设。评选“茂名十大美丽旅游乡村”。

　　推动农业产业化发展。积极培育家庭农场、农业龙头企业等新型经营主体，新打造一批年产值超10亿元农业领军企业。加快电白省级农产品加工示范园和高州现代农业园区建设。支持粤西农批、农村电商加快发展。落实粮食安全责任制。

　　抓好精准扶贫工作。把精准扶贫与产业发展有机结合，整合扶贫资金支持有前景的产业项目。完成5.3万人脱贫任务。推进180条省定贫困村创建新农村示范村。实施农村贫困户危房改造8058户。推进农村留守儿童、妇女、老人“三关爱”工作。扎实做好与广西南宁、来宾“携手奔小康”扶贫协作工作。

　　（七）大力加强文化引领，打造“好心茂名”城市形象

　　打造城市文化品牌。制定实施《茂名市冼夫人文化发展纲要》，在城市建设中厚植地方文化元素。深入开展道德模范、身边好人推荐评议活动，建立善行义举榜、好人榜。打造一批核心价值观主题公园，创建一批核心价值观学校示范点。

　　大力发展文化旅游产业。积极推进高州和化州冼太纪念园、冼太夫人文化旅游景区、信宜南方碧玉商贸城、1959文化创意街等重点文化产业项目建设。抓好南粤古驿道保护利用。以重大文体休闲活动为媒介，推动文化传播、旅游推介、遗产保护共同发展。

　　加快发展文体事业。规划建设奥体中心“一场两馆”，支持电白建设“四馆一中心”。大力提高竞技体育水平。加大公共体育场馆免费低收费开放力度。启动改造茂名影剧院。建成开放露天矿博物馆。完成村（社区）综合性文化服务中心建设任务。组织开展“唱响茂名”等群众性文化活动。筹办南国书香节茂名分会场暨茂名书展，倡导全民阅读。精心打造体育节、徒步节、马拉松、龙舟邀请赛、市长杯足球赛等群众体育品牌活动，引导健康生活方式。

　　（八）大力推进绿色发展，加强生态文明建设

　　推进生态建设工程。深入开展新一轮绿化茂名大行动，努力创建国家森林城市。完成森林碳汇工程3.65万亩，新建森林公园1个、湿地公园1个，升级提升森林公园27个、湿地公园4个，建设乡村绿化美化示范点128个。继续建设银杏景观大道。推进露天矿生态公园绿化优化提升。推进大型人工鱼礁示范和美丽海湾试点。

　　加强环境综合整治。持续推进中央环保督察和海洋督察反馈问题整改。完善重污染天气应急协调机制。深入实施河长制，建立湖长制。编制鉴江流域和小东江流域系统水质提升方案。加快推动彭村湖黄竹河综合治理与生态修复。完成污水处理设施和配套管网建设任务。抓好黑臭水体整治。实施近岸海域污染防治，推进电白区关屋河、寨头河、森高河3条入海河流消除劣Ⅴ类水质工作。推进农业面源污染防治。加强土壤污染防治、辐射、固体废物等管理。稳步推进县域绿能环保发电项目。

　　加强环保源头治理。做好生态红线划定工作。加强自然资源资产审计。推动信宜、化州产业转移园申报省园区循环化改造示范区。推进循环经济示范中心项目建设。发展环保产业推动环保治理，借助市场化力量推进污水处理、垃圾处理、农村环境治理。

　　（九）大力提升基本公共服务均等化水平，加强和创新社会治理

　　扎实推进教育创现工作。推动五区（市）通过广东省推进教育现代化先进县（市、区）达标验收。全力推进广东石油化工学院、广东农林科技职业学院、广东茂名幼儿师范专科学校、广州科技职业技术学院滨海校区等新建（扩建）项目建设，全面建成“一本五专”高校。加大学前教育和特殊教育投入。

　　大力促进就业。探索“校企双制”和“产教融合”人才培养模式。全年新增城镇就业6万人，促进创业2200人，转移劳动力技能培训1万人，城镇登记失业率控制在3.5%以内。

　　加快卫生创强步伐。推进14家县级公立医院升级、“5+3”中心卫生院升级、村卫生站公建规范化等项目建设。加快医联体建设和家庭医生式服务签约工作。提高基层医院服务能力，推进中医药事业发展。加强精神卫生建设。完善计生服务管理。

　　巩固提升民生保障水平。加大社保扩面征缴工作力度。探索推进医保城乡一体化改革，稳步推进机关事业单位养老保险制度改革和医保支付方式改革。保持房地产市场平稳健康发展。开工建设棚户区改造住房495套，建成各类保障性住房891套。

　　切实加强社会治理。加快建设立体化社会治安防控体系，深化治安重点地区整治，严厉打击黄赌毒、盗抢骗、黑拐枪等违法犯罪活动。深入推进全民禁毒工程。突出抓好交通秩序整治。坚决防范和遏制重特大安全生产事故，开工建设石化产品运输车辆综合服务中心，力争建成危化品应急救援基地。加强社会组织培育发展。着力构建清朗网络空间。强化食品药品安全治理。抓好殡葬改革工作。切实做好统计、工青妇、民族宗教、国防动员、科普、外事、侨务、双拥、三防、人防、气象、地震、残疾人、档案、红十字、方志文史等工作。

　　各位代表，打铁必须自身硬。茂名振兴发展的每一步，绝不是轻轻松松、敲锣打鼓就能实现的，必须以过硬作风锤炼过硬素质，打造重实干、求实绩的机关作风。把作风建设的成效体现到提振信心上来。把精力和心思放在谋发展、促发展上，在推动发展上多干事、干成事，在干事创业的成就感中树立信心。把作风建设的成效体现到担当作为上来。紧盯重点工作和重要事项，发扬“钉钉子”、“拧螺丝”精神，坚持不懈抓落实；支持在职责范围内大胆决策，善于调动各方力量，集中攻关，全力突破。把作风建设的成效体现到改善营商环境上来。立足让企业满意、让群众满意，突破制约招商引资和项目落地的关键点、梗阻点，提升政务服务质量效率。把作风建设的成效体现到提升群众获得感上来。保持党同人民群众的血肉联系，增强群众观念和群众感情，全力为群众办实事、办好事。切实加强政府系统党风廉政建设，担负起全面从严治党主体责任，履行“一岗双责”，推动各级领导干部知责明责、守责担责。着力消除权力寻租空间，遏制重点领域腐败。坚持不懈反对“四风”，贯彻落实中央八项规定实施细则和省委、市委要求。扎实开展“不忘初心、牢记使命”主题教育和“大学习、深调研、真落实”活动。加强民主法治建设，自觉接受人大及其常委会监督，认真落实其各项决议、决定并定期报告工作。自觉接受人民政协民主监督，接受审计和舆论监督。办理好人大代表建议和政协委员提案。加强法治政府建设，提高依法行政水平。

　　各位代表！新思想引领新时代，新目标开启新征程。让我们紧密团结在以习近平同志为核心的党中央周围，以习近平新时代中国特色社会主义思想为指导，在省委、省政府和市委正确领导下，不忘初心、牢记使命，以务实作风和奋斗姿态推动各项工作争先晋位，努力在粤东西北振兴发展中继续走在前列。
